# A 9-10-Bit Adjustable and Energy-Efficient Switching Scheme for Successive Approximation Register Analog-to-Digital Converter with One Least Significant Bit Common-Mode Voltage Variation

**DOI:** 10.3390/s24113273

**Published:** 2024-05-21

**Authors:** Yunfeng Hu, Chaoyi Chen, Lexing Hu, Qingming Huang, Bin Tang, Mengsi Hu, Bingbing Yuan, Zhaohui Wu, Bin Li

**Affiliations:** 1 School of Electronics and Information Engineering, University of Electronic Science and Technology of China, Zhongshan Institute, Zhongshan 528402, China; 2 School of Microelectronics, South China University of Technology, Guangzhou 510640, China

**Keywords:** biosensor, energy-efficient, SAR ADC, 9-10-bit adjustable

## Abstract

A 9-10-bit adjustable and energy-efficient switching scheme for SAR ADC with one-LSB common-mode voltage variation is proposed. Based on capacitor-splitting technology and common-mode conversion techniques, the proposed switching scheme reduces the DAC switching energy by 96.41% compared to the conventional scheme. The low complexity and the one-LSB common-mode voltage offset of this scheme benefit from the simultaneous switching of the reference voltages of the capacitors corresponding to the positive array and the negative array throughout the entire reference voltage switching process, and the reference voltage of each capacitor in the scheme does not change more than two voltages. The post-layout result shows that the ADC achieves the 54.96 dB SNDR, the 61.73 dB SFDR, and the 0.67 μw power consumption with the 10-bit mode and the 48.33 dB SNDR, the 54.17 dB SFDR, and the 0.47 μw power consumption with the 9-bit mode in a 180 nm process with a 100 kS/s sampling frequency.

## 1. Introduction

Biosensors are capable of generating individual life parameters in real time, with high chip size and power consumption requirements due to their portability and high endurance. The analog-to-digital converter (ADC) is one of the core modules of the electronic terminal equipment; it can realize the conversion of analog signals to digital signals. The successive approximation analog-to-digital converter (SAR ADC) is suitable for low power consumption applications due to its simple structure, high speed, and low power consumption [1,2,3,4,5]. This design proposes a 9-10-bit adjustable scheme and designs a bit-count control circuit [6]. Based on the selection of the bit-count mode, the number of capacitors and registers involved in the conversion is controlled to reduce the number of bits in the ADC in scenarios where high precision is not required; the number of capacitors is known intuitively; thus, the overall circuit power consumption is reduced.

In the energy consumption analysis of the SAR ADC, it was learned that the capacitor array DAC consumed about 30% of the overall energy consumption [7,8,9], while the simulation energy analysis of the chip found that the DAC’s energy consumption was closer to 70% of the total energy consumption [10,11,12], coupled with the fact that it was relatively difficult to improve the structure of the analog module circuit. 

It can be seen that the enhancement of the capacitive array DAC switching scheme can reduce the overall energy consumption of the chip. Compared with the conventional capacitive array DAC structure [13], the energy consumption of the set-and-down DAC structure can be reduced by 81.26% [14]; that of the C-2C common-mode voltage DAC structure can be reduced by 90.61% [15]; that of the three reference voltages, an additional reference voltage, *V*_cm_, and the switching scheme (tri-level) DAC structure can be reduced by 96.89% [16]; that of the common-mode voltage monotonic (VMS) DAC structure can be reduced by 97.66% [17]; that of a perfect application of *V*_cm_ and the monotonic technique (hybrid) DAC structure can be reduced by 98.83% [18]; and that of the capacitor-splitting structure, charge-average switching technique, and *V*_aq_ (equal to *V*_ref_/4) (VQS) DAC structure can be reduced by 98.10% [19], and two sub-capacitor arrays with the common-mode DAC structure (TSC) can be reduced by 98.45% [20].

These DAC schemes greatly reduce the energy consumption of the DAC structure, but these structures include at least three reference voltages, which increase the overall circuit complexity of the SAR ADC and thus the energy consumption of the other modules; they even cause a common-mode voltage shift [14,15,16,17,18,19]. Compared to [20], the proposed scheme does not require a third reference voltage and is 9-10-bit adjustable. In order to reduce the overall circuit complexity and power consumption of the SAR ADC, a low-complexity capacitor array DAC switching scheme with one-LSB common-mode voltage variation for SAR ADC was designed; it applies bridge switches and the floating technique to reduce DAC switching energy. The reference voltage of the capacitors corresponding to the positive array and the negative array is simultaneously changed, except for the last voltage variation. Additionally, the reference voltage of each capacitor in the scheme is transformed by no more than two voltages. This approach exhibits the characteristics of having no dependency on an extra reference voltage and of having a one-LSB common-mode voltage offset. Finally, the SAR ADC circuits are simulated and analyzed using a 180 nm CMOS process. The post-layout result shows that the ADC achieves the 54.96 dB SNDR, the 61.73 dB SFDR, and the 0.67 μw power consumption with the 10-bit mode and the 48.33 dB SNDR, the 54.17 dB SFDR, and the 0.47 μw power consumption with the 9-bit mode.

## 2. Design of the Proposed SAR ADC

**Figure 1:**
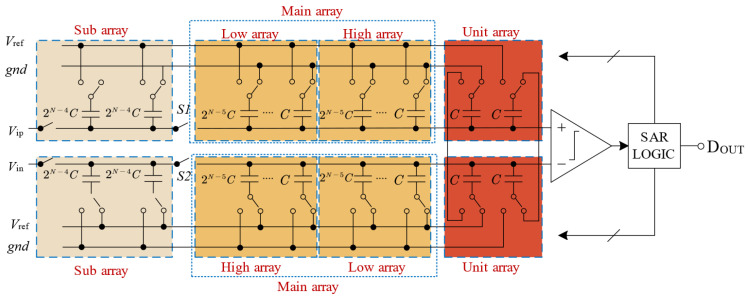
The proposed architecture of N-bit SAR ADC.

The N-bit SAR ADC of the structure is shown in Figure 1. The SAR ADC consists of the DAC, SAR logic, comparator [21], and bootstrapped sample switch. The DAC consists of the sub-array, main array, and unit array. The main array consists of the high array and low array.

### 2.1. DAC Switching Scheme

**Figure 2:**
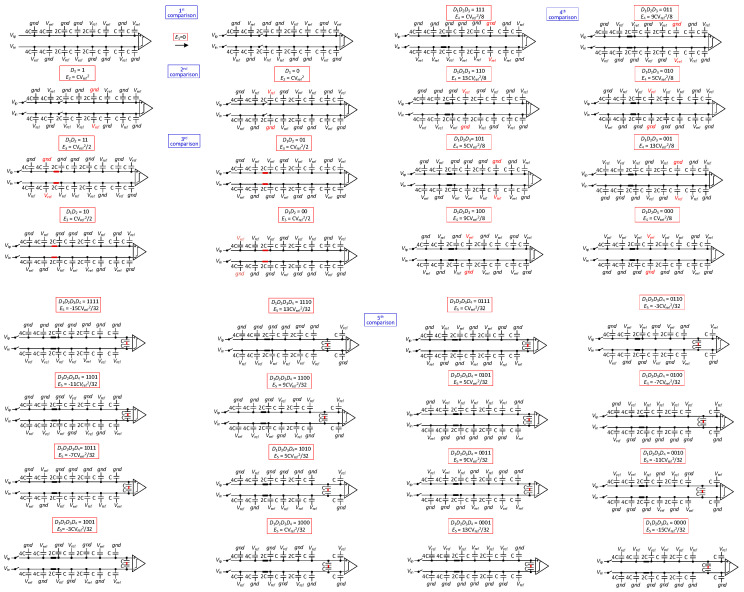
The first five steps of the 6-bit switching scheme.

**Figure 3:**
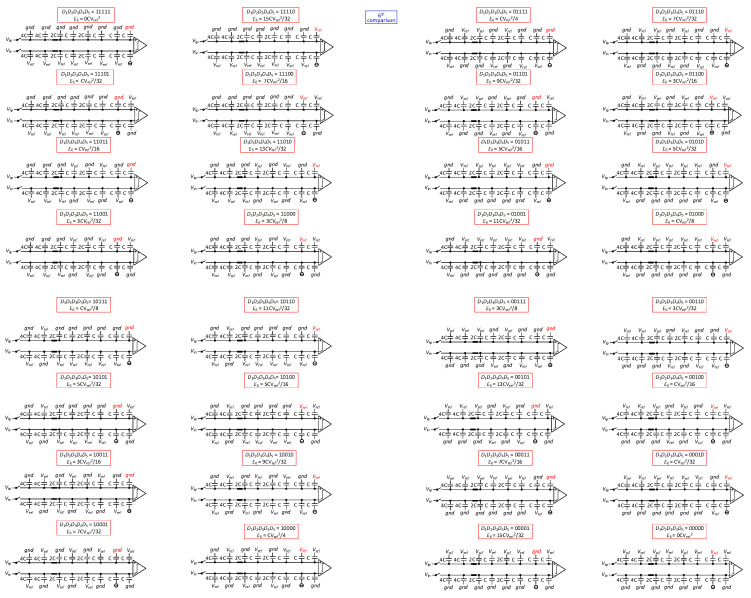
The sixth step of the 6-bit switching scheme.

The proposed SAR ADC operates in five phases, as shown in Figure 2 and Figure 3. To illustrate the working principle of SAR ADC, a 6-bit SAR ADC conversion diagram is shown below:

Phase 1: The input signal is sampled on the top plates of all the capacitors by the sampling switch. The bottom plates of the capacitors of the high array are connected to $V_{ref}$, and the bottom plates of the capacitors of the low array are connected to gnd. One of the bottom plates of the sub-capacitor arrays is connected to $V_{ref}$, and the other is connected to gnd. One of the bottom plates of the unit capacitor arrays is connected to $V_{ref}$, and the other is connected to gnd. After sampling, the sampling switch is turned off. The comparator then performs the first comparison and outputs the result of the first comparison (*D*_1_), without consuming any switching energy.

Phase 2: Based on the previous output of the comparator, the bottom plate of the corresponding capacitor (2^N-5^C) of the high array on the high-voltage side is switched from $V_{ref}$ to gnd, while the bottom plate of the corresponding capacitor (2^N-5^C) of the low array on the other side (low-voltage side) is switched from gnd to $V_{ref}$, and the other arrays remain unchanged. As a result, the voltage on the high-voltage side decreases $V_{ref}/4$, while the voltage on the low-voltage side increases $V_{ref}/4$. The comparator then performs the second comparison and outputs the result of the second comparison (*D*_2_).

(1) $$E\left(2\right)=2^{N-6}CV_{ref}^{2}$$

Phase 3: According to the results of the first and the second comparisons, the DAC varies the reference voltage of the corresponding capacitors. When *D*_1_*D*_2_ is 11, the capacitance bottom plate connected to $V_{ref}$ of the sub-capacitor array in the positive-phase capacitance array is switched to gnd, and the capacitance bottom plate connected to the gnd of the sub-capacitor array in the reversed-phase capacitance array is switched to $V_{ref}$; when *D*_1_*D*_2_ is 00, the capacitance bottom plate connected to the gnd of the sub-capacitor array in the positive-phase capacitance array is switched to $V_{ref}$, and the capacitance bottom plate connected to $V_{ref}$ of the sub-capacitor array in the reversed-phase capacitance array is switched to gnd; when *D*_1_*D*_2_ is 10 or 01, the reference voltage of the sub-capacitor array stays unchanged. After the changes in the reference voltage, the switches S1, S2 close; then, the comparator performs the comparison and outputs the result of the third comparison (*D*_3_).

(2) $$E\left(3\right)=2^{N-7}CV_{ref}^{2}$$

Phase 4: After the completion of the previous comparison, the bottom plate of the corresponding capacitor in the high array on the high-voltage side is switched from $V_{ref}$ to gnd, while the bottom plate of the corresponding capacitor in the low array on the other side (low-voltage side) is switched from gnd to $V_{ref}$, and the other arrays remain unchanged, e.g., in the fourth comparison, the second largest capacitor in the high array on the high-voltage side is switched from $V_{ref}$ to gnd, while the second largest capacitor in the low array on the other side (low-voltage side) is switched from gnd to $V_{ref}$, and the other arrays remain unchanged. During the entire reference voltage switching process, the reference voltage of the capacitor corresponding to the positive array and the reversed array converts at the same time, regardless of which side is changing. The reference voltage switching scheme makes the common-mode voltage variation, and the reference voltage transformation of each capacitor in this scheme is not more than two, which reflects the low complexity and zero common-mode voltage offset characteristics of the scheme.

The ADC repeats the process until the (*N* − 1)th comparison is completed. The common-mode voltage remains constant during the switching process. The DAC switching energy for each comparison from the fourth comparison to the (*N* − 1)th comparison is

(3) $$E\left(i\right)=\left\{\begin{array}{c} 2^{N-2i-1}\cdot \left(1-2D_{i-1}\right)\cdot \left(2D_{i-1}-1\right)\\ +2^{N-4-i}\cdot \left[3-2\left(2D_{1}+D_{2}\right)\right]\cdot \left(2D_{i-1}-1\right)\\ +\sum_{j=6}^{i}2^{N-j-i+1}\cdot \left(2D_{i-1}-1\right)+2^{N-\left(i+2\right)} \end{array}\right\}CV_{ref}^{2}$$

Phase 5: In the (*N* − 1)th comparison, depending on the result of the (*N* − 2)th comparison, the bottom plate of the capacitor connected to $V_{ref}$ of the unit capacitor array on the higher side of the voltage is connected to the bottom plate of the capacitor connected to the gnd of the unit capacitor array on the lower side of the voltage, and the other capacitors is kept unchanged. In the *N*th comparison, the capacitor array switching energy is

(4) $$E\left(n-1\right)=\left\{\begin{array}{c} 2^{-3}\cdot \left(3-2\left(2D_{1}+D_{2}\right)\right)\cdot \left(2D_{n-2}-1\right)+\left(2-4D_{n-2}\right)\cdot \left[\frac{2D_{n-2}-1}{2^{n}}\right]\\ +\sum_{j=6}^{N}2^{-j+2}\cdot \left(1-2D_{j-2}\right)\cdot \left(2D_{n-2}-1\right) \end{array}\right\}CV_{ref}^{2}$$

Phase 6: In the *N*th comparison, depending on the result of the (*N* − 1)th comparison, based on the transformation in phase 5, the two connected unit capacitors are disconnected, and the unit capacitor belonging to the negative array is suspended. When the voltage of the positive array is higher than that of the negative array, the unit array belonging to the positive array is connected to *gnd*; conversely, the unit capacitor belonging to the positive array is connected to *V_ref_*. In the *N*th comparison, the capacitor array switching energy is

(5) $$E\left(n\right)=\left\{\begin{array}{c} 2^{-3}\cdot \left(3-\left(2D_{1}+D_{2}\right)\right)\cdot \left(2D_{n-1}-1\right)+\sum_{j=6}^{N}2^{-j+2}\cdot \left(1-D_{j-3}\right)\cdot \left(2D_{n-1}-1\right)\\ +\frac{1}{2}\cdot \left(1-D_{n-1}\right)+\left(2-D_{n-2}-D_{n-1}\right)\cdot \left[\left(2D_{n-1}-1\right)\frac{1}{2^{n-1}}\right] \end{array}\right\}CV_{ref}^{2}$$

For the *N*-bit resolution, the average switching energy of the capacitor array switching energy is:

(6) $$E_{average}=\left[\begin{array}{c} 2^{N-6}+2^{N-7}-2^{-N}+\left(\frac{1}{4}-2^{-N}\right)\\ +\sum_{i=4}^{N-2}2^{N-i-1}\cdot \left(2^{-1}-2^{-i}\right) \end{array}\right]CV_{ref}^{2}$$

### 2.2. Bootstrapped Sample Switch

**Figure 4:**
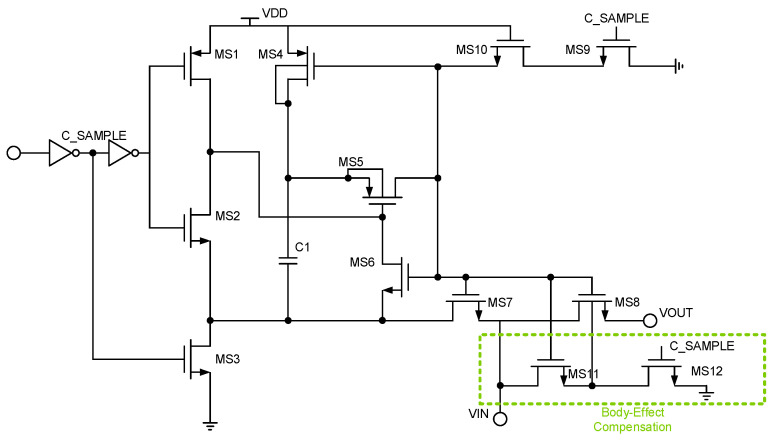
Bootstrapped sample switch with body effect compensation.

The bootstrapped sample switch is a very important part of the SAR ADC, as shown in Figure 4; here, the design of the overall simulation allows sufficient design margins in the original gate bootstrapped sample switch on the basis of the substrate bias effect of the increase in the body effect compensation technology [22]. It is easy to see from Equation (7), for the impact of *R*_on_, in addition to the *V*_gs_, that the impact of the threshold voltage is not a small proportion of *R*_on_.

(7) $$R_{on}=\frac{1}{\frac{\partial I_{D}}{\partial V_{DS}}}=\frac{1}{\mu_{n}C_{ox}\frac{W}{L}\left(V_{gs}-V_{TH}-V_{DS}\right)}$$

**Figure 5:**
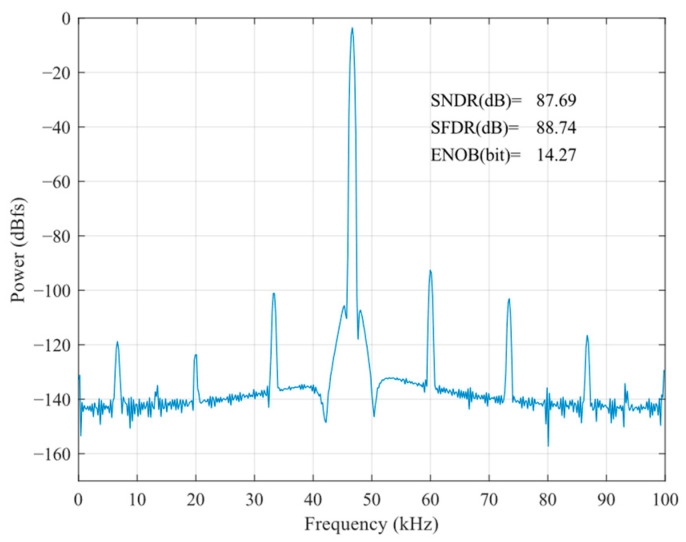
Post-layout result of the bootstrapped sample switch.

**Figure 6:**
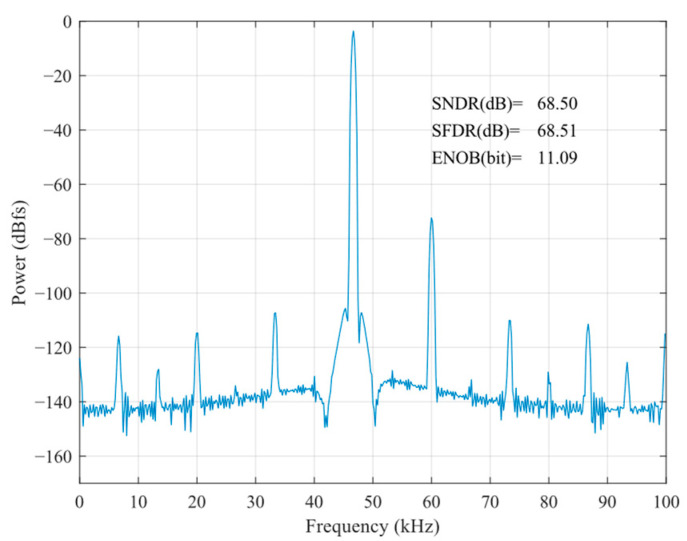
Post-layout result of the bootstrapped sample switch without body effect compensation.

According to Equation (7), in the sampling stage, the substrate end of the sampling MOSFET can be connected to *V*_in_ to make *V*_S_ = *V*_B_, which, to a certain extent, reduces the nonlinear factor of *R*_on_ resistance brought about by the threshold voltage. The post-layout result of the bootstrapped sample switch is shown in Figure 5 and Figure 6, which illustrate the post-layout result of the bootstrapped sample switch without body effect compensation. It is easy to see that the body effect compensation technique makes the effective number of bits of the bootstrapped sample switch 3.18 bits higher.

(8) $$V_{TH}=V_{S}+V_{FB}\pm k{\left[\pm \left(2\phi_{FB}+V_{S}-V_{B}\right)\right]}^{1/2}+2\phi_{FB}$$

### 2.3. 9-10-Bit Adjustable SAR Logic

**Figure 7:**
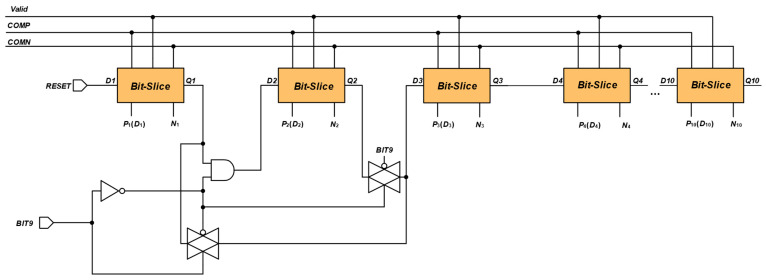
9-10-bit adjustable SAR logic based on dynamic logic.

As shown in Figure 7, the 10-bit SAR circuit has 10 dynamic logic units by default. When the SAR ADC works in a 9-bit mode, the logic requirements of the SAR logic need to shield a single dynamic logic unit; at the same time, from the point of view of saving power consumption, the second logic unit is shielded off, so that the switches S1, S2 are disconnected; this not only achieves the logic requirements, but also reduces the overall capacitance, so as to save energy consumption. In this paper, the number of bits of the circuit is controlled by setting the BIT9.

## 3. Simulation Results and Discussion

**Figure 8:**
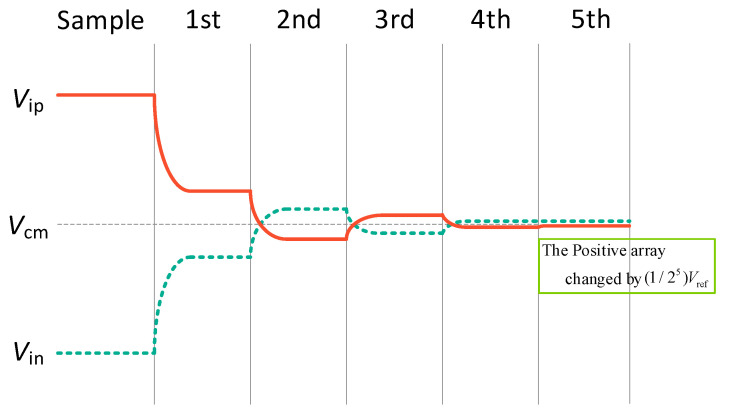
Waveform of the proposed 6-bit switching scheme.

The voltage variation in the 6-bit scheme is shown in Figure 8, where the first four voltage variations are common-mode transformations, and the (1/2*^n^*^−1^)*V_ref_* common-mode shift is caused by the last voltage variation, with *n* equal to 6.

**Figure 9:**
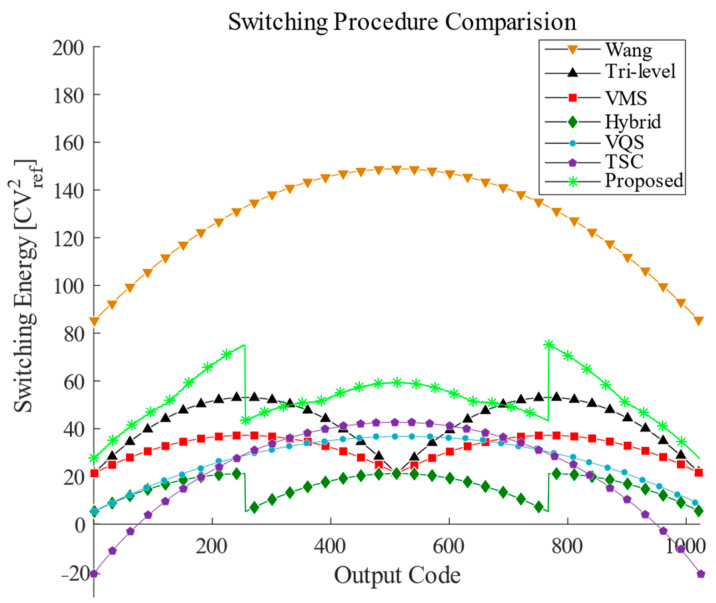
Switching energy against output codes.

The successive approximation waveform of the proposed switching scheme without common-mode voltage variation is shown in Figure 8. Compared with the conventional switching scheme, the average switching energy is reduced by 96.14%. The switching energy at each output code for the different switching schemes is shown in Figure 9. The comparison of several switching schemes for the 10-bit SAR ADC is shown in Table 1. Although VMS [17] and the hybrid [18] are more energy-efficient, they have high logic complexity and a large common-mode voltage offset. While VQS [19] obtains better energy savings as well as area reduction at a low common-mode voltage offset, two additional reference voltages are introduced.

**Figure 10:**
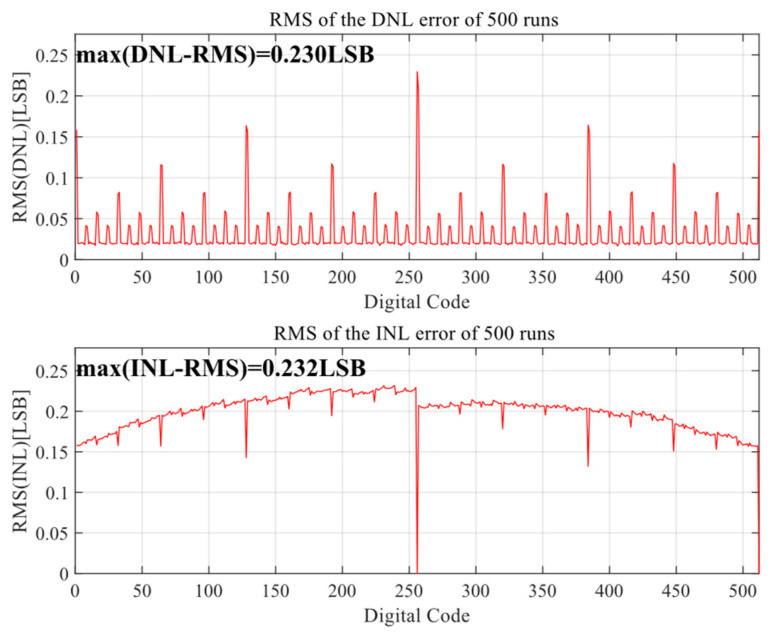
INL and DNL of DAC for 9-bit mode.

**Figure 11:**
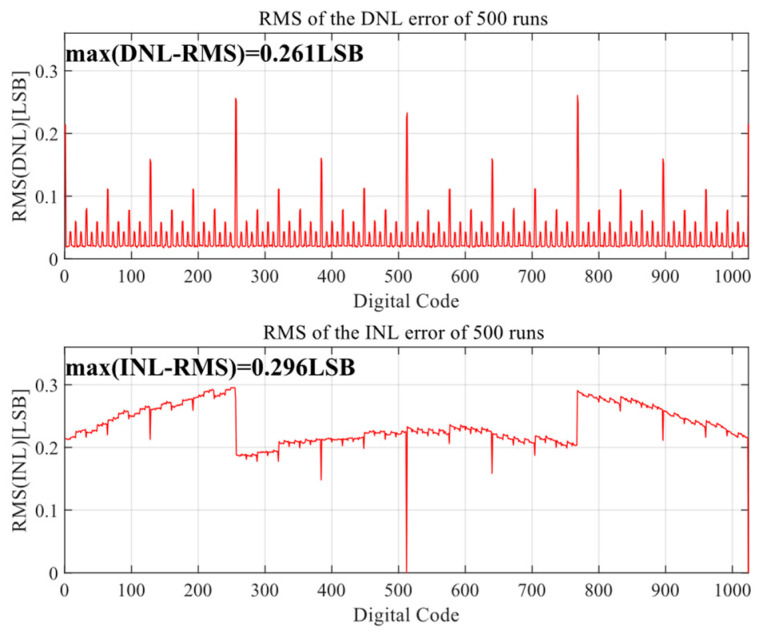
INL and DNL of DAC for 10-bit mode.

Figure 10 and Figure 11 show the Monte Carlo analysis results of the proposed DAC switching scheme after 500 simulations. When the unit capacitance mismatch is σ_u_/C_u_ = 1%, the RMS DNL and RMS INL of the proposed DAC switching scheme are 0.261 LSB and 0.296 LSB, respectively, for the 10-bit mode and 0.230 LSB and 0.232 LSB for the 9-bit mode, meeting the requirement that the ADC nonlinear error should be less than 0.5 LSB.

**Figure 12:**
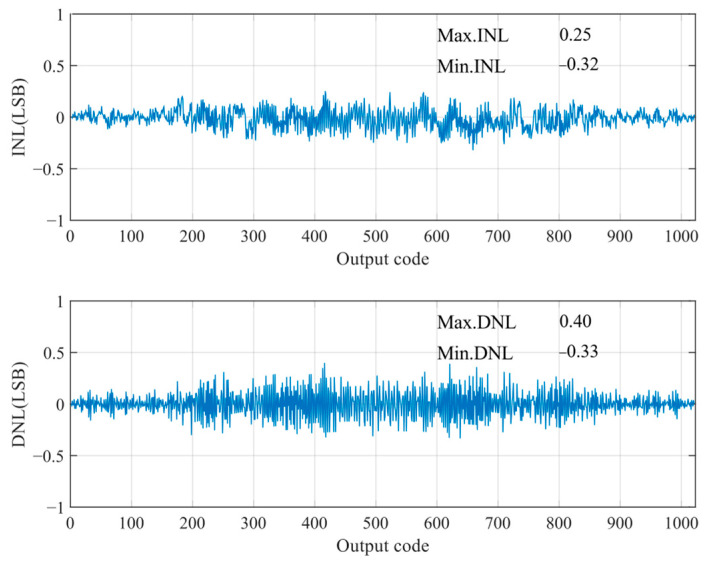
INL and DNL of SAR ADC.

**Figure 13:**
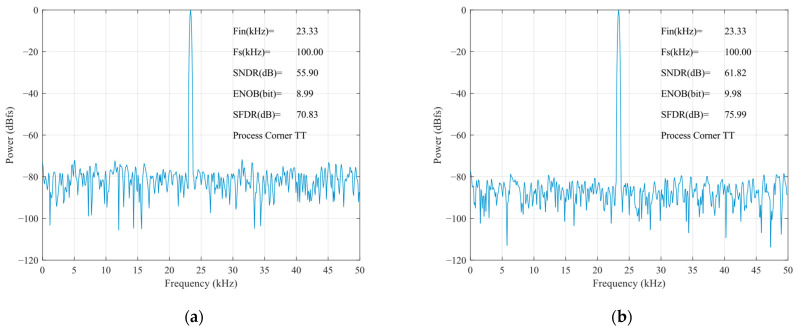
Simulated results of FFT for SAR ADC: (**a**) 9-bit mode; (**b**) 10-bit mode.

**Figure 14:**
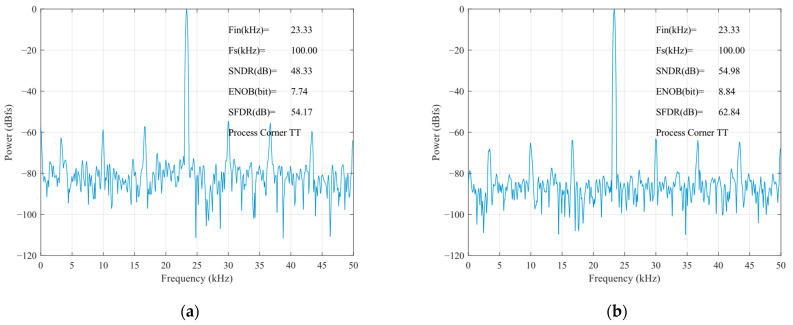
Post-layout results of FFT for TT corner: (**a**) 9-bit mode; (**b**) 10-bit mode.

**Figure 15:**
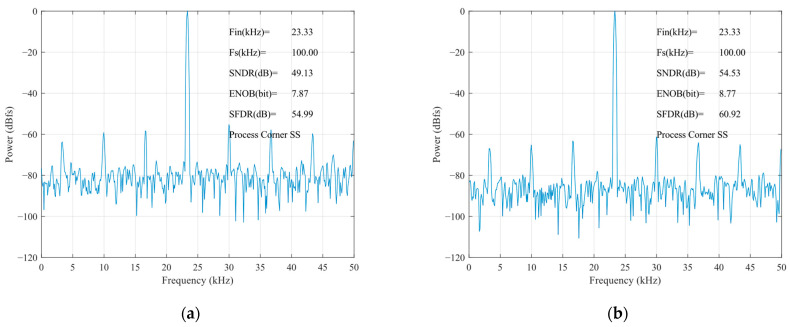
Post-layout results of FFT for SS corner: (**a**) 9-bit mode; (**b**) 10-bit mode.

**Figure 16:**
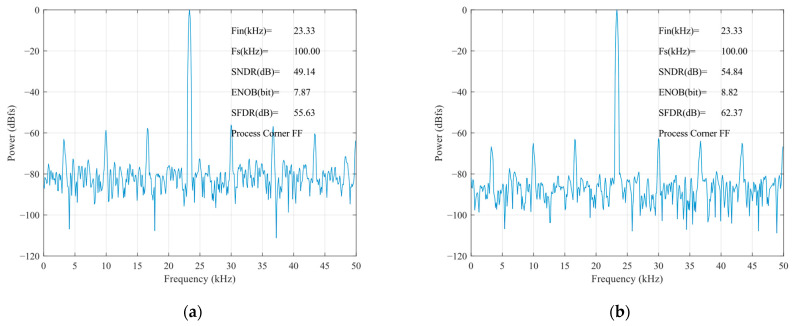
Post-layout results of FFT for FF corner: (**a**) 9-bit mode; (**b**) 10-bit mode.

**Figure 17:**
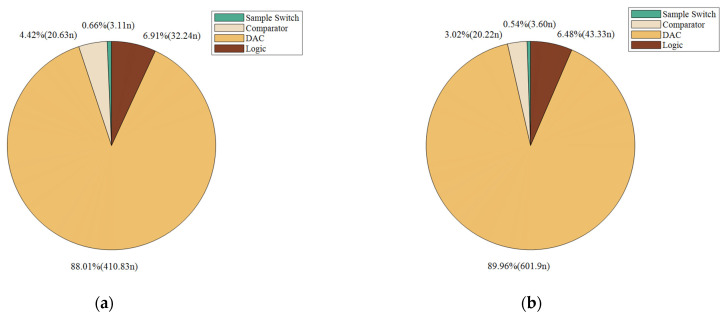
Power consumption of proposed SAR ADC with 10-bit mode: (**a**) 9-bit mode; (**b**) 10-bit mode.

**Figure 18:**
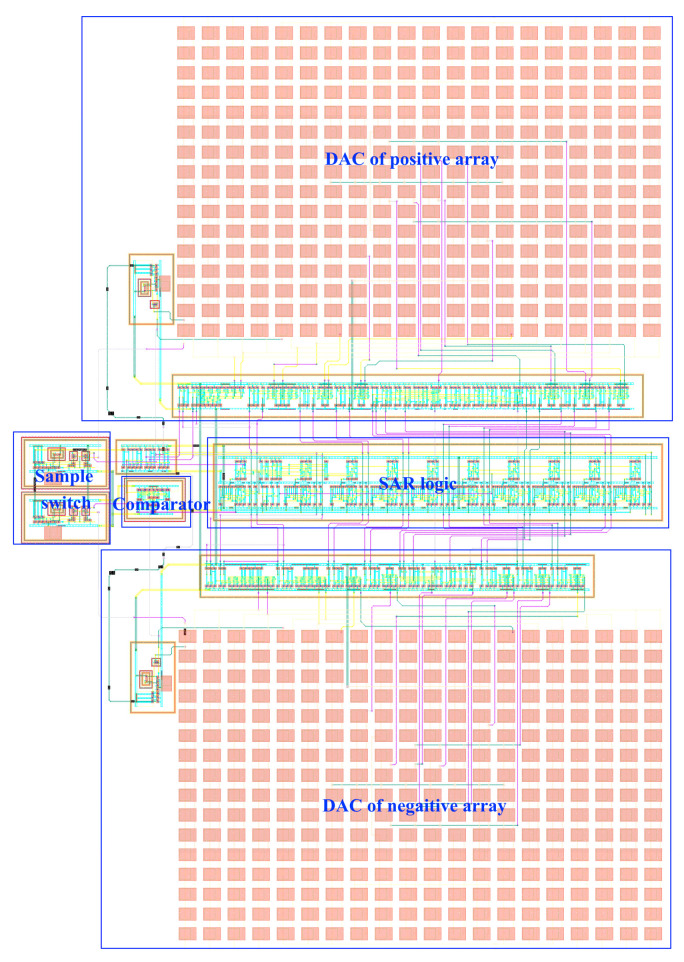
Layout design of proposed SAR ADC.

The static parameters of the SAR ADC were tested using the code density test method with an input sinusoidal signal frequency of 23.33 kHz, a unit capacitance value of 37.8 fF for the capacitance array, and a sampled signal frequency of 100 kHz. The differential nonlinearity (DNL) and integral nonlinearity (INL) are shown in Figure 12. The INL is −0.32 LSB ~ +0.25 LSB (LSB is the lowest significant bit), the DNL is −0.33 LSB ~ 0.40 LSB, and both the INL and the DNL are less than 0.5 LSB, so, the designed circuit meets the requirements of the static characteristics. Figure 13 shows the simulated results of the proposed SAR ADC for the 9-bit mode and 10-bit mode. The ADC achieves a 61.82 dB signal-to-noise and distortion ratio (SNDR), 9.98 ENOB, and a 55.90 dB signal-to-noise and distortion ratio (SNDR), 8.99 ENOB. Figure 14 shows the post-layout result, which has extracted the parasitic resistance and the parasitic capacitance of the proposed SAR ADC for the 10-bit mode and 9-bit mode. The ADC achieves a 54.96 dB signal-to-noise and distortion ratio (SNDR), 8.84 ENOB, and a 48.33 dB signal-to-noise and distortion ratio (SNDR), 7.74 ENOB. The performance comparison of various ADCs [12,19,23,24] is shown in Table 2. Figure 15 and Figure 16 show the results of this ADC in the 9-bit and 10-bit mode with different process corners of the post-layout simulation. Also, the pie chart of the overall energy consumption of the ADC in the 10-bit mode is shown in Figure 17. The layout design is shown in Figure 18, and its overall occupied area is 360 μm × 520 μm.

## 4. Conclusions

This paper presented a 9-10-bit adjustable and energy-efficient switching scheme SAR ADC with a one-LSB common-mode voltage variation. The proposed SAR ADC consists of the DAC, dynamic comparator, bootstrapped sample switch, and SAR logic. The DAC consists of a positive capacitor array and a negative capacitor array, and each array is composed of the same three sub-capacitor arrays. 

Based on the capacitor-splitting and common-mode conversion techniques, the proposed switching scheme reduces the energy consumption by 96.41% compared to the conventional scheme. The simultaneous switching of the reference voltages of the capacitors throughout the entire reference voltage switching process and each capacitor having only two reference voltages accomplished the low complexity and zero common-mode voltage variation in the proposed scheme. In addition, the post-layout results show that the ADC achieves the 54.96 dB SNDR, the 61.73 dB SFDR, and the 0.67 μw power consumption with the 10-bit mode and the 48.33 dB SNDR, the 54.17 dB SFDR, and the 0.47 μw power consumption with the 9-bit mode. The FoM of the proposed SAR ADC with the 10-bit mode is 14.5 fJ/conv.-step, and with the 9-bit mode, it is 21.9 fJ/conv.-step.

## Figures and Tables

**Table 1:** Comparison of energy saving and common-mode shift for different switching schemes of a 10-bit SAR ADC.

Switching Scheme	Average Energy $\left({CV_{ref}}^{2}\right)$	Energy Saving	Number of Reference Voltages	Area Reduction	Common-Mode Shift
Conventional [13]	1363.3	Reference	2	Reference	0
Set and Down [14]	255.5	81.26%	2	50%	512 LSB
Wang [15]	128	90.61%	3	75%	512 LSB
Tri-level [16]	42.42	96.89%	3	75%	256 LSB
VMS [17]	31.88	97.66%	3	75%	256 LSB
Hybrid [18]	15.88	98.83%	3	75%	384 LSB
VQS [19]	26.58	98.10%	4	87.5%	1 LSB
TSC [20]	21.08	98.45%	3	75%	1 LSB
Proposed	52.58	96.14%	2	75%	1 LSB

**Table 2:** Performance comparison.

Parameter	[19] *	[20] *	[23]	[12]	[24]	This Work **
Process (nm)	180	180	180	180	180	180
Resolution (bits)	10	10	10	10	10	9/10
Sampling Rate (kS/s)	20	1000	200	200	200	100
Supply Voltage (V)	0.6	1.5	0.6	0.6	0.6	1
ENOB (bits)	9.4	9.69	9.3	9.08	9.16	7.74/8.84
DNL (LSB)	−0.569/0.572	−0.30/0.33	−0.26/0.29	−0.32/0.30	−0.21/0.27	−0.33/0.4
INL (LSB)	−0.422/0.533	−0.20/0.36	−0.80/0.36	−0.56/0.38	−0.45/0.43	−0.32/0.25
Power Consumption (µW)	0.042	10.45	2.01	1.01	1.76	0.47/0.67
FoM (fJ/conv. Step)	3.11	12.65	15.51	9.32	15.38	21.9/14.5

* Simulated results. ** Post-layout results.

## Data Availability

Data are contained within the article..
